# Co-design of eHealth Interventions With Children and Young People

**DOI:** 10.3389/fpsyt.2018.00481

**Published:** 2018-10-18

**Authors:** Hiran Thabrew, Theresa Fleming, Sarah Hetrick, Sally Merry

**Affiliations:** ^1^Department of Psychological Medicine, University of Auckland, Auckland, New Zealand; ^2^School of Health, Victoria University of Wellington, Wellington, New Zealand

**Keywords:** co-design, eHealth, research, children and adolescents, method, agile methodology, kanban, scrum

## Abstract

Co-design, defined as collective creativity across the entire design process, can lead to the development of interventions that are more engaging, satisfying, and useful to potential users. However, using this methodology within the research arena requires a shift from traditional practice. Co-design of eHealth interventions with children and young people has additional challenges. This review summarizes the applied core principles of co-design and recommends techniques for undertaking co-design with children and young people. Three examples of co-design during the development of eHealth interventions (Starship Rescue, a computer game for treating anxiety in children with long-term physical conditions, a self-monitoring app for use during treatment of depression in young people, and HABITS, the development of an emotional health and substance use app, and eHealth platform for young people) are provided to illustrate the value and challenges of this contemporary process.

## Introduction

### What is co-design?

Co-design originated from the field of participatory design (1) and involves a process of collective creativity applied across the *entire* design process (2). Co-design is different from co-creation, defined as *any* act of collective creativity (2). During co-design, active collaboration occurs between researchers, designers, developers, and users as “experts of their experiences” (3). Co-design involves more than participants ***saying*** what they want from interventions or services and being observed ***doing*** (to see how they use interventions or services); it involves jointly exploring and articulating needs and jointly exploring and ***making*** solutions (4). The term “co-design” is sometimes incorrectly applied to include processes, more correctly termed “consultation” or “engagement” during which users views on a need, idea, or product are sought in a limited manner. Co-design aims for better design based on a richer, deeper understanding of what users know, feel, and even dream.

### Stages of co-design

Most co-design processes begin with understanding the current needs and behaviors of users, developing concepts that are tested in simple, fast, and low-cost ways before being improved through an iterative process. Two structured methods applicable to service design and research are the five stage process by Bowen et al. (5) and the six stage process by Boyd et al. (6). Bowen proposes (i) understanding and sharing experiences, (ii) exploring blue-sky ideas, (iii) selecting and developing blue-sky concepts, (iv) converging to practical proposals, and (v) prototyping and evaluating. Similarly, Boyd recommends (i) engagement, (ii) planning, (iii) exploring, (iv) developing, (v) deciding, and (vi) changing. What may be new for many academics is the “scoping” phase of design. Rather than beginning with acquired expert knowledge or academic research, co-designers seek to deeply understand contemporary lived experience from users to know what problems need to be solved.

### Agile design

**Figure 1:**
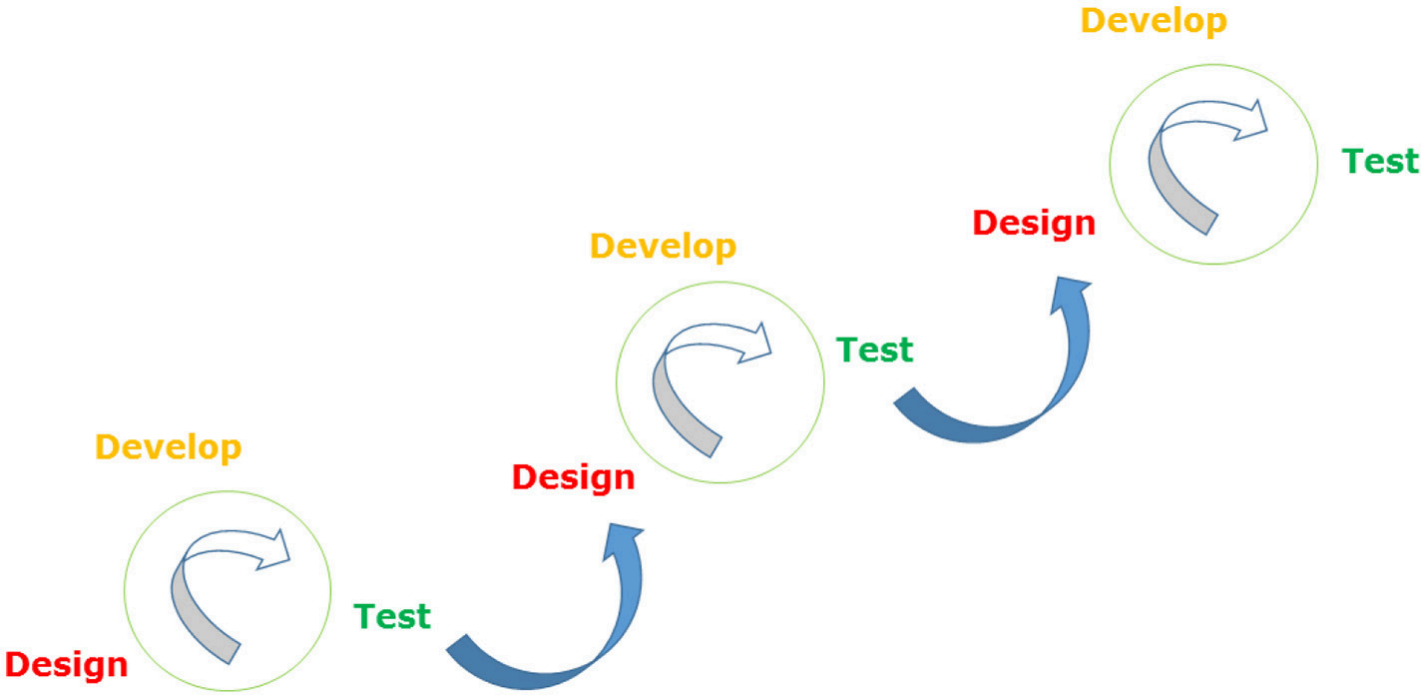
The agile method.

The pace of co-design can be increased using agile design. This involves discovering, designing, developing and testing a product or service in a series of (often rapid) cycles known as “sprints” (Figure 1). “Scrum” methodology and “kanban” methodology are two forms of agile design. Both involve regular meetings between a pre-determined set of people so that sprints can be kept on track. A typical scrum includes a product owner (the person with vision and authority), a scrum master (a facilitator and advisor, who removes impediments to progress, but does not manage the team), and a team (usually 3–10 members, matched to the needs of the project, who are self-managing and jointly responsible for delivering the product via a series of tasks). During scrum-based design, work is done within predetermined timeframes (usually 2–4 weeks), with a deliverable product at the end of each sprint. There is an emphasis on cross-functionality and no one within the main team has specified roles (www.cprime.com/resources/what-is-agile/what-is/scrum). Kanbans do not involve fixed-length sprints or regular team meetings. Instead, tasks are pulled from a backlog and product is released as it is created, allowing for continuous delivery. Team members can choose tasks aligned with their area of expertise. However, over-specialization can reduce team effectiveness (www.atlassian.com/agile/kanban).

### Co-design and research

**Figure 2:**
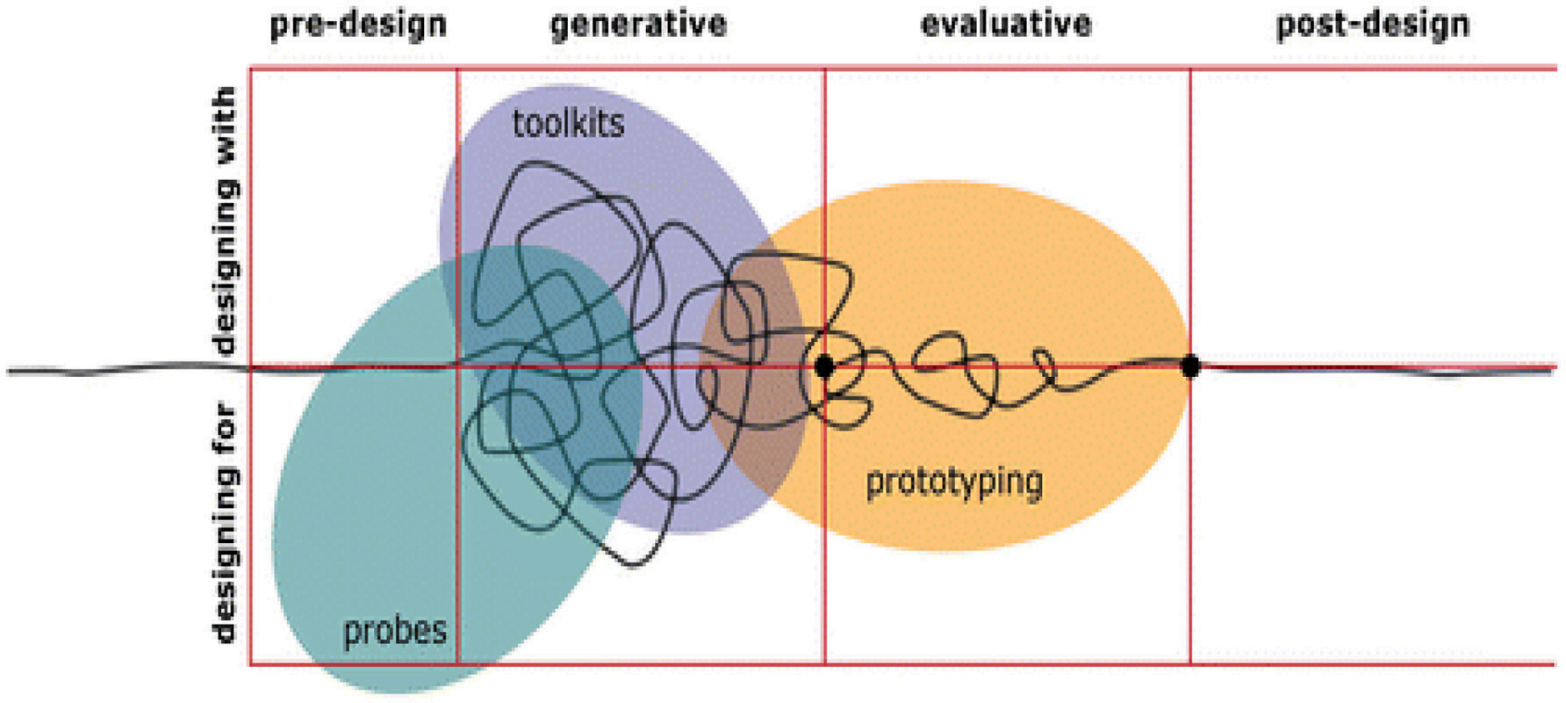
Use of probes, toolkits, and prototypes across different stages of research [reproduced with permission from Sanders and Stappers (7)].

Co-design can be employed during generative, pre/post-test and evaluative research (2) and during the complete development of an intervention. During generative research, it can help to produce ideas and concepts to be designed and developed and help to identify what will actually be useful and usable in the future. During pre/post-test research, it can help researchers gain a better understanding of people's experiences in the context of their past, present and future lives. As part of evaluative research, it can help to assess the effectiveness of existing products, spaces, systems, or services. When used across the complete development of a new intervention, co-design usually begins with the use of superficial ***probes*** to engage users, moves on to more intensive generation of ideas using detailed ***toolkits***, narrows into the development and evaluation of ***prototypes*** and ends with more targeted post-design evaluation (Figure 2). During each stage, researchers, designers, and users may have differing levels of involvement in the process, but all remain part of the team.

### Practical techniques for facilitating co-design

Numerous creative techniques exist for making users' experiences available for discussion. These include the use of visual materials, story-telling, fun and playful activities, and the physical creation of products. It is important to embrace ambiguity to create a space that fosters creativity and supports reflection (www.smallfire.co.nz). Materials that can support such processes with participants of all ages include whiteboards, collages, storyboards, inspiration cards, modeling tools (Play-Doh, Lego, Meccano, etc.), and games (8). Prototypes have a special place in the process of co-design. As less abstract and more “visible” entities created during the co-design process, they encourage more focused engagement, discussion, and testing of hypotheses. They allow people to experience a product or situation that did not previously exist and facilitate the consideration of multiple, potentially overlapping, theories or perspectives (9). To achieve the best results, it is important to engage users early, ensure there is a representative spread of users (not just the easy to reach), and have staff (researcher/designer) buy-in and willingness to go beyond the comfort zone of a tightly controlled research process and environment (6). Studio design (http://jpattonassociates.com/wp-content/uploads/2015/03/design_studio_quickref.pdf) is a specific process that can be used to create and critique multiple versions or prototypes of proposed interventions in the space of a few hours. This process of “ideation,” rather than “iteration,” leads to the generation of a wider range of possible solutions from which the design team can choose the most viable.

### Benefits of co-design

Whether it is used for research or service improvement, co-design can have benefits for projects, users and services (10). Projects may be enhanced by the range of available ideas, a better understanding of user needs, and reduction in development costs and time. Users may benefit by receiving a more satisfactory intervention or higher quality of service and becoming educated about future interventions (11). Services can improve their relationships with users, focus on user needs and can increase the likely support and success of innovations (12). However, to date, there is limited research regarding the effectiveness of products or services that have been co-designed compared with those that have not been co-designed (13, 14).

### Challenges of co-design

Potential downsides of co-design include challenges navigating ethics and review board application processes where committees expect pre-planned processes and unfamiliar with the flexibility required in co-designed research (15). This can be managed by upfront explanation of the co-design process and face to face discussion with ethics committees. Frustration regarding the pace and scale of change is common, particularly for users (5). So, being clear about how long the process will take and what is within the brief are important. Conflict and tension relating to issues of power may occur within co-design groups. This is particularly likely if users have had previous poor relationships with researchers or clinicians (16). Although it may be tempting to exclude “difficult” users from the process, it is also important to address their needs if an intervention is likely to be useful to its intended audience. Importantly, diverse users may have different needs and preferences. For example, young people who are not distressed are likely to find helping-seeking easier than those with depression (17). Furthermore, even within seemingly like groups, preferences can be diverse and conflicting. Where multiple types of user are engaged, care needs to be taken to balance their needs or to meet the needs of some users well. Trying to please everyone with one design can lead to the creation of a universally unsatisfactory hybrid or “duck-horse” (http://www.creativevisualart.com). In such situations, it may be more useful to segment an audience and interventions (https://www.thecompassforsbc.org/how-to-guides/how-do-audience-segmentation). Project and funding creep are extremely likely unless careful thought has been put into managing these risks. Defining the type, extent and realistic parameters associated with the co-design process (including funding limitations) is vital to achieving a timely and satisfactory result. Finally, co-design processes do not inherently lead to the creation of an outcome that will always be generalizable across user groups, time and place. One of the maxims of agile design is “fail quickly” (https://www.digitalartsonline.co.uk/features/creative-business/embrace-failure/). This refers to the concept that not all seemingly excellent, well thought out ideas will succeed in practice. While rapid testing and iteration is important, so is ceasing work or changing direction when a prototype is not working well. This can be challenging in a publically funded research setting. Even where a product does not fail, its appeal may not generalize to broad target groups. Robust co-design processes can identify these differences in scoping or understanding phases and can test concepts early with diverse groups, including users that are introduced at later stages.

### Co-design with children and young people

Undertaking co-design with children and young people broadly follows the principles outlined above. In addition, it is important to make participatory workshops engaging and productive (18). Ways of doing so include the use of comfortable and age-appropriate environments (ideally not a clinic or research office), meaningful ice-breakers and familiar games (don't reinvent the wheel). The use of naturally occurring groups (e.g., friends or classmates) may assist conversation, but less so when sensitive topics are being discussed. Young people may be motivated by cash rewards or being named as “authority figures” in the process (19). For participants with lower motivation, the use of short activities, clearly stated outputs, concrete examples and familiar situations can all help (20). Culturally-relevant concepts (such as metaphors connected with youth or particular cultures) and the use of settings and props to communicate cultural references can improve engagement (5). The ideal duration of workshops for children and young people is usually between 30 min and 2 h, depending on the age and abilities of the audience. Safety and wellbeing is important, so regular toilet breaks, the availability of snacks or “brain food” and sensitivity to participants' mental health issues can improve the group's productivity. As stated above, it is important to source a representative range of users and not just those who are keen to participate in research. Finally, a useful (and potentially anxiety-provoking) mantra for running genuinely responsive co-design workshops is “make a plan, then throw it away” (www.wearesnook.com).

### Co-design and eHealth

Co-design of eHealth interventions has added complexity relating to the multi-faceted interaction between researchers, users, and software developers. The overarching co-design philosophy may need to be underpinned by conscious co-design relationships between researchers and users, software developers and users, and researchers and software developers. In addition, although eHealth interventions designed for independent access may only have one set of users, interventions designed for supported or clinic-based use may also need to cater to the needs of support people and clinicians to optimize their uptake and adherence. Finally, due to the rapidly changing digital landscape, updates and re-iterations of successfully co-designed eHealth interventions should be anticipated (21, 22).

### Three examples of co-design during the development of eHealth interventions for children and young people

#### Example 1: development of starship rescue, a computer game for treating anxiety in children with long-term physical conditions

Children and young people with long-term physical conditions have higher rates of psychological issues, especially anxiety during the co-design process of an eHealth intervention to address anxiety, seven focus groups (three for children and parents, two for adolescents, one for pediatricians and one for general practitioners) were undertaken using a semi-structured format and clips of existing eHealth interventions. Five key themes emerged from this scoping phase: (1) the experience of long term physical conditions as an anxiety provoking journey; (2) limited access to information and eHealth-related interventions to support this journey; (3) desires for interventions that assist with multiple aspects of the illness experience, especially anxiety; (4) diversity of preferences regarding the format and vehicle of such interventions (many, but not everyone, wanted games); and (5) the importance of trust regarding the source of interventions.

During the second phase, therapeutic and educational components for the game including CBT, biofeedback and the use of a bi-centric frame of reference were identified by the researcher (HT). Game design principles, flow methodology, and Bartle gamer profiles were contributed by a game designer, and biofeedback expertise was provided by a biofeedback specialist. User-identified preferences, including the use of a versatile gaming format and story, were incorporated into an initial prototype. An agile design process (Figure 1) was undertaken at this stage to develop, test and refine individual modules of the intervention in 2–3 sprints over a 6 months period. User feedback was incorporated to address technical issues, make instructions clearer, and develop the look and feel of the game. An open trial of the prototype will be undertaken later this year.

#### Example 2: development of a self-monitoring app for use during treatment of depression in young people

Young people with depression commonly experience suicidal ideation and engage in self-harm (23) and require support to monitor symptoms outside of face-to-face treatment (24). During a scoping/generative phase (Figure 2), SH and colleagues engaged young people with lived experience of self-harm individually to understand their needs. Young people described relationship difficulties, distressing emotions, a sense of isolation, exposure to other's self-harm, comparing themselves to others, and school/work difficulties as factors likely to lead to self-harm. Factors mitigating the risk of self-harm were different for everyone and environmentally dependent. Distraction, access to immediate support and environmental modification were identified as helpful techniques.

Four co-design workshops, using a design studio (25) approach in the context of a overarching participatory design framework (12), were subsequently conducted with young people to develop an app to meet their needs (26). Individual designs were critiqued by peers with a focus on goals rather than what was liked/not liked (i.e., providing two to three ways it solves a problem and one to two ways it could be improved). Wireframes of optimal features were included in a prototype app. Key features included a customizable mood monitoring feature (with a positive focus and avoiding typical emotional labels and simplistic “emotion faces”); a one-touch function to access real-life support, “distractions,” immersive activities designed to distract them from acute distress, and a personalized “care package” of activities designed to enhance well-being (e.g., playlists, relaxation activities, inspirational quotes, supportive messages from friends, photographs or videos reminding them of fun times, and links to funny YouTube videos).

#### Example 3: the development of HABITS, a multistage digital intervention for youth mental health

New Zealand adolescents, particularly those of Maori and Pacific ethnicities, have high rates of depression, suicidality and poor rates of health care access (27). Mainstream approaches, not targeted to these user groups, have had limited impact. TF and colleagues developed a multistage co-design process, beginning with an extended scoping/generative phase (Figure 2) with Maori, Pacific, and other young people, both with and without experience of mental distress. Aims of this phase included the identification of current eHealth-related needs, behaviors, preferences, and opportunities, without presupposing specific goals or interventions.

Eight focus groups [two whanau (extended family) groups, one school group and three community groups], and a brief online survey (*n* = 74) were undertaken over a 6 months period. Information was obtained about personal preferences regarding health-related and non-health related websites and apps as well as online behaviors and help-seeking strategies, particularly when feeling down, depressed, or struggling with mental health issues. There were two profoundly important findings. Firstly, participants were open to receiving online support. Many participants said they spent time online when distressed. When directly mental health related, it almost always involved posting about their situation or distress on social media accounts with varying levels of anonymity. Almost none had sought help from strangers nor looked for information or help online other than by posting on social media. Where people did seek help this usually occurred when they were “desperate,” such as during a suicidal crisis. Secondly, adolescents with similar levels of mental health need and backgrounds had diverse preferences. Although some considered that a gamified, playful interface was important for engagement and that early intervention approaches would be the most desirable and powerful, others considered that a serious, to the point, simple interface was essential and that games would be trivializing.

During a reflective “targeting” stage, this information was triangulated with known population health needs, cultural, clinical, and researcher advice, themes from the literature, available resources, implementation opportunities and ongoing user input. It was agreed that any digital intervention to support wellbeing in this group could not rely on individual help-seeking and would require “push” approaches or outreach systems. In addition, for pragmatic reasons, a decision was made to prioritize the needs of younger adolescents who were interested in earlier intervention and often preferred non-threatening, game-based approaches. Finally, engagement was undertaken with education stakeholders to align planned online interventions with existing school-based screening and healthcare. These critical shaping decisions were reviewed and reflected on with stakeholders prior to the commencement of a formal co-design development phase (currently underway). From this point, two initial interventions to address emotional health and substance use are being developed and will be refined and tested during the coming year.

## Conclusion

Co-design is an important step for increasing the extent to which interventions are user-centered. Although it is likely that co-designed interventions and services are more engaging, more satisfying, and or more useful to potential users, there are challenges in undertaking research using co-design and the relative effectiveness of co-designed interventions has only been researched to a limited extent. Co-design can successfully be undertaken with children and young people. However, additional thought needs to be given to settings and techniques to ensure meaningful engagement and participation from these groups. Our experience suggests that alongside other necessary developments in the field (21), co-design has promise for increasing the impact of eHealth interventions for children and young people. Co-design is a process not a product.

## Author contributions

HT, TF, SM, and SH conceptualized and contributed substantial content to the paper. All co-authors approved the paper.

### Conflict of interest statement

The authors declare that the research was conducted in the absence of any commercial or financial relationships that could be construed as a potential conflict of interest.

## References

[1] Bjerknes G Ehn P Kyng P Computers and Democracy. Aldershot: Avebury (1987).

[2] Sanders EBN Stappers PJ Co-creation and the new landscapes of design. CoDesign (2008) 4:5–18. 10.1080/15710880701875068

[3] Sleeswijk Visser F Stappers PJ Van der Lugt R Sanders EBN Contextmapping: experiences from practice. CoDesign (2005) 1:119–49. 10.1080/15710880500135987

[4] Sanders EBN From user-centred to participatory design approaches. In: Frascara J editor. Design and the Social Sciences: Making Connections. London: Taylor and Francis (2002). p. 1–8.

[5] Bowen S Sustar H Wolstenholme D Dearden A. Engaging teenagers productively in service design. Int J Child Comp Interact. (2014) 1:71–81. 10.1016/j.ijcci.2014.02.001 26516621 PMC4579821

[6] Boyd H McKernon S Mullin B Old A. Improving healthcare through the use of co-design. N Z Med J. (2010) 125:4–15. 22854362

[7] Sanders EBN Stappers PJ Probes, toolkits and prototypes: three approaches to making in codesigning. CoDesign (2014) 10:5–14. 10.1080/15710882.2014.888183

[8] Gray D Brown S Macanufo J Gamestorming: A Playbook for Innovators, Rulebreakers, and Changemakers. Boston: O'Reilly Media, Inc (2010).

[9] Stappers PJ Prototypes as central vein for knowledge development. In: Paper Presented at Proto:Type 2010 (Dundee) (2014).

[10] Steen M Manschot M De Koning N Beneits of co-design in service design projects. Int J Design (2011) 5:53–60.

[11] Alam I An exploratory investigation of user involvement in new service development. J Acad Market Sci. (2002) 30:250–61. 10.1177/0092070302303006

[12] Hagan P Collins P Metcalf A Nicholas M Rahilly K Swainston N Participatory Design of Evidence-Based Online Youth Mental Health Promotion Intervention and Treatment: a Young and Well Cooperative Research Centre Innovative Methodologies Guide. Melbourne, VIC: Young and Well Cooperative Research Centre (2012).

[13] Mulvale A Miatello A Hackett C Mulvale G Applying experience-based co-design with vulnerable populations: lessons from a systematic review of methods to involve patients, families and service providers in child and youth mental health service improvement. Pat Exp J. (2016) 3:117–29

[14] Richard L Piper DA Weavell W Callander R Iedema R Furler J Advancing engagement methods for trials: the CORE study relational model of engagement for a stepped wedge cluster randomization controlled trial of experience-based co-design for people living with severe mental illnesses. Trials (2017) 18:169 10.1186/s136063-017-1878-7 28388937 PMC5385022

[15] Goodyear-Smith F Jackson C Greenlagh T. Co-design and implementation research: challenges and solutions for ethics committees. BMC Med. Ethics (2015) 16:78. 10.1186/s12910-015-0072-2 26573410 PMC4647576

[16] Robert G Cornwell J Locock L Purushotham A Sturmey G Gager M. Patients and staff as codesigners of healthcare services. Br Med J. (2015) 350:g7714. 10.1136/bmj.g7714 25670179

[17] Carlton PA Deane FP. Impact of attitudes and suicidal ideation on adolescents' intentions to seek professional psychological help. J Adolesc. (2000) 23:35–45. 10.1006/jado.1999.0299 10700370

[18] Pedersen J Buur Games and movies: towards innovative co-design with users. In: Scrivener SAR Ball LJ Woodcock A editors. Collaborative Design: Proceedings of Codesigning. London: Springer (2000). p. 93–100.

[19] Glasemann M Kanstrup AM Evoking creativity: young diabetics design their own mobile diabetes supporter. In: Research Symposium. Aarlborg (2008).

[20] Mazzone E Read J Beale R Understanding children's contributions during informant design. In: Proceedings of the 22nd British HCI Group Annual Conference on People and Computers: Culture, Creativity, Interaction-Volume 2. Liverpool: BCS Learning and Development Ltd (2008). p. 61–4.

[21] Fleming TM De Beurs D Khazaal Y Gaggioli A Riva G Botella C. Maximizing the impact of e-therapy and serious gaming: time for a paradigm shift. Front Psychiatry (2016) 7:65. 10.3389/fpsyt.2016.00065 27148094 PMC4834305

[22] de Beurs D van Bruinessen I Noordman J Friele R van Dulmen S Active involvement of end users when developing Web-based mental health interventions. Front Psychiatry (2017) 8:72 10.3389/fpsyt.2017.00072 28515699 PMC5413572

[23] Cash SJ Bridge JA. Epidemiology of youth suicide and suicidal behavior. Curr Opin Pediatr. (2009) 21:613–19. 10.1097/Mop.0b013e32833063e1 19644372 PMC2885157

[24] Hetrick SE Goodall J Yuen HP Davey CG Parker AG Robinson J Comprehensive online self-monitoring to support clinicians to manage risk of suicide in youth depression: a pilot study. Crisis (2017) 38:147–57. 10.1027/0227-5910/a000422 27659516

[25] Warfel TZ Prototyping: a Practitioner's Guide. New York, NY: Rosenfeld Media (2009).

[26] Hetrick SE Robinson J Burge E Blandon R Mobiliio B Rice SM Youth co-design of a mobile phone app to facilitate self-monitoringQ18 and management of mood symptoms in young people with major depression, suicidal ideation and self-harm. JMIR Ment Health (2017) 5:e9. 10.2196/mental.9041 PMC5801516 29362208

[27] Fleming T Clark TC Denny S Bullen P Crengle S Peiris-John R. Stability and change in the mental health of New Zealand secondary school students 2007–2012: results from the national adolescent health surveys. Aust N Z J Psychiatry (2014) 48:472–80. 10.1177/0004867413514489 24317154

